# Determining Effects of Non-synonymous SNPs on Protein-Protein Interactions using Supervised and Semi-supervised Learning

**DOI:** 10.1371/journal.pcbi.1003592

**Published:** 2014-05-01

**Authors:** Nan Zhao, Jing Ginger Han, Chi-Ren Shyu, Dmitry Korkin

**Affiliations:** 1 Informatics Institute, University of Missouri, Columbia, Missouri, United States of America; 2 Department of Computer Science, University of Missouri, Columbia, Missouri, United States of America; 3 Bond Life Science Center, University of Missouri, Columbia, Missouri, United States of America; University College London, United Kingdom

## Abstract

Single nucleotide polymorphisms (SNPs) are among the most common types of genetic variation in complex genetic disorders. A growing number of studies link the functional role of SNPs with the networks and pathways mediated by the disease-associated genes. For example, many non-synonymous missense SNPs (nsSNPs) have been found near or inside the protein-protein interaction (PPI) interfaces. Determining whether such nsSNP will disrupt or preserve a PPI is a challenging task to address, both experimentally and computationally. Here, we present this task as three related classification problems, and develop a new computational method, called the SNP-IN tool (non-synonymous SNP INteraction effect predictor). Our method predicts the effects of nsSNPs on PPIs, given the interaction's structure. It leverages supervised and semi-supervised feature-based classifiers, including our new Random Forest self-learning protocol. The classifiers are trained based on a dataset of comprehensive mutagenesis studies for 151 PPI complexes, with experimentally determined binding affinities of the mutant and wild-type interactions. Three classification problems were considered: (1) a 2-class problem (strengthening/weakening PPI mutations), (2) another 2-class problem (mutations that disrupt/preserve a PPI), and (3) a 3-class classification (detrimental/neutral/beneficial mutation effects). In total, 11 different supervised and semi-supervised classifiers were trained and assessed resulting in a promising performance, with the weighted f-measure ranging from 0.87 for Problem 1 to 0.70 for the most challenging Problem 3. By integrating prediction results of the 2-class classifiers into the 3-class classifier, we further improved its performance for Problem 3. To demonstrate the utility of SNP-IN tool, it was applied to study the nsSNP-induced rewiring of two disease-centered networks. The accurate and balanced performance of SNP-IN tool makes it readily available to study the rewiring of large-scale protein-protein interaction networks, and can be useful for functional annotation of disease-associated SNPs. SNIP-IN tool is freely accessible as a web-server at http://korkinlab.org/snpintool/.

## Author Summary

Many genetic diseases in humans and animals are caused by combinations of single-letter mutations, or SNPs. When these mutations occur in a protein-coding region of a genome, they can have a profound effect on the protein's function and ultimately on a health-related phenotype. Recently, a growing number of evidence suggests that many of SNPs reside on or near the protein regions that are required for the interactions with other proteins. Some of these SNPs could rewire the protein-protein interactions altering the functions of the protein interaction complexes, while other SNPs are neutral to the interactions. Understanding the effect of SNPs on the protein-protein interactions is a challenging problem to solve, both experimentally and computationally. Here, we leverage the machine learning methods by training a computational predictor to tell apart the mutations that are harmful to protein-protein interactions from those ones that are not. We use these tools in two case studies of mutations affecting the protein-protein interaction networks centered around the genes associated with breast cancer and diabetes.

This is a *PLOS Computational Biology* Methods article.

## Introduction

Being one of the most prevalent types of genetic variation in humans, single nucleotide polymorphisms (SNPs) occur in both coding and non-coding regions of the genome and have been associated with a number of Mendelian diseases and complex genetic disorders [1], [2]. With the rapid advancement of DNA sequencing and genotyping technology, millions of SNPs have been determined [3], [4]. An average gene is estimated to have several non-synonymous missense SNPs (nsSNPs), each substituting an amino acid residue [5]. Nevertheless, our knowledge of SNPs that cause a disease is very limited. Understanding whether or not a mutation or a group of mutations induce changes of a molecular function is often the first step towards finding the missing link between the genetic variation and the disease.

Recent studies of disease networks have linked many nsSNPs with protein-protein interactions [6], [7]. Understanding how these mutations can rewire the interaction network mediated by proteins associated with the disease is critical in studying complex genetic disorders, such as cancer, autism, and diabetes [8]–[10]. Unfortunately, the interaction landscape determined by the genetic variants of the disease-associated genes is far from being fully reconstructed. Thus, computational methods can play an important role in modeling nsSNP-induced rewiring of a disease network.

The growing interest in understanding the relationship between a genetic variation and its functional effect on a protein has lead to a number of recent *in-silico* methods. A group of methods introduced the idea of computational mutagenesis to study the structure-function relationship [11], predict the changes in enzyme activity [12], [13], detect disease potential of a SNP [14], and characterize other functional effects [15]. Most recently, a number of computational alanine scanning methods were developed to study protein-protein interactions (PPIs) and protein-peptide interactions [16]. These methods aimed at finding residues in the interaction interface that would disrupt the interaction when mutated to alanine; they did it by estimating the relative free energy change (ΔΔ*G*) between the wild-type and mutant PPI complexes. Another group of methods focused on predicting the effects of general nsSNPs on protein function and distinguishing them from functionally neutral mutations [17]–[30]. Finally, several works studied the effects of disease-associated nsSNPs on protein-protein interactions by investigating the changes in binding energy using force field and electrostatic calculations [31], [32] and understanding the structural effects caused by nsSNPs that lead to the disruption of PPI [6], [33]. However, in spite of the tremendous progress, developing an accurate approach that predicts the effect of an nsSNP on the protein function, including protein-protein interaction, remains an open problem.

The goal of this paper is to introduce a novel computational approach for the characterization of effects on PPIs caused by nsSNPs (nsSNP-induced effects). The idea of our approach is to consider prediction of such effects as a classification problem. Specifically, we defined three related classification problems that differ in the available input information and the types of nsSNP-induced effects to be identified and characterized. Leveraging the machine learning methodology, we formulated each of the three problems as the supervised and semi-supervised learning tasks. The comparative assessment of the independently built classifiers using a variety of the supervised and semi-supervised methods has demonstrated feasibility of the machine learning approach in addressing each of the above problems.

## Methods

**Figure 1:**
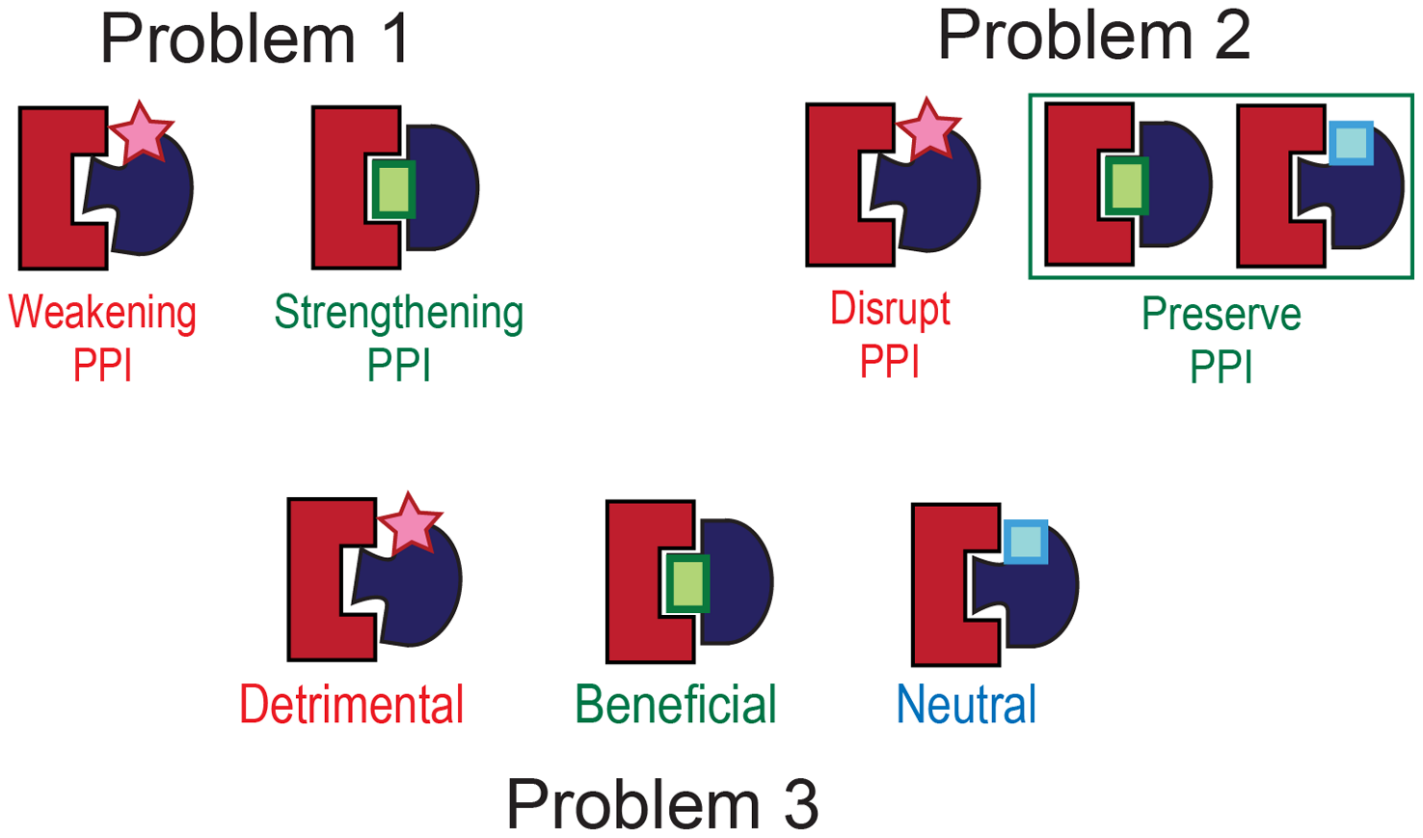
Three related classification problems addressed in this work. Classes in the 3-class problem are used to form the 2-class problems.

The problem of determining whether an nsSNP within a gene has any effect on a PPI mediated by the gene product is broken down into three related classification problems (Fig. 1). In the first problem, we assume that it is known that an nsSNP affects a biochemical function mediated by a PPI. Such a functional change may be a result of the nsSNP disrupting the interaction or, on the contrary, significantly increasing the binding affinity, which may cause for a transient complex to become permanent. Therefore, our goal in the first problem is to determine whether the nsSNP has a *strengthening or weakening* effect on the PPI. The second problem is to determine whether an nsSNP is likely to *disrupt* or *preserve* a PPI, without any prior knowledge on changes in the biochemical function mediated by the interaction. Finally, the third, most challenging, problem is to predict whether an nsSNP has one of three effects on a PPI, *detrimental*, *neutral*, or *beneficial*, again without any prior knowledge of the functional changes associated with the PPI Thus, the first and second problems are formulated as 2-class problems, and the third one as a 3-class problem.

**Figure 2:**
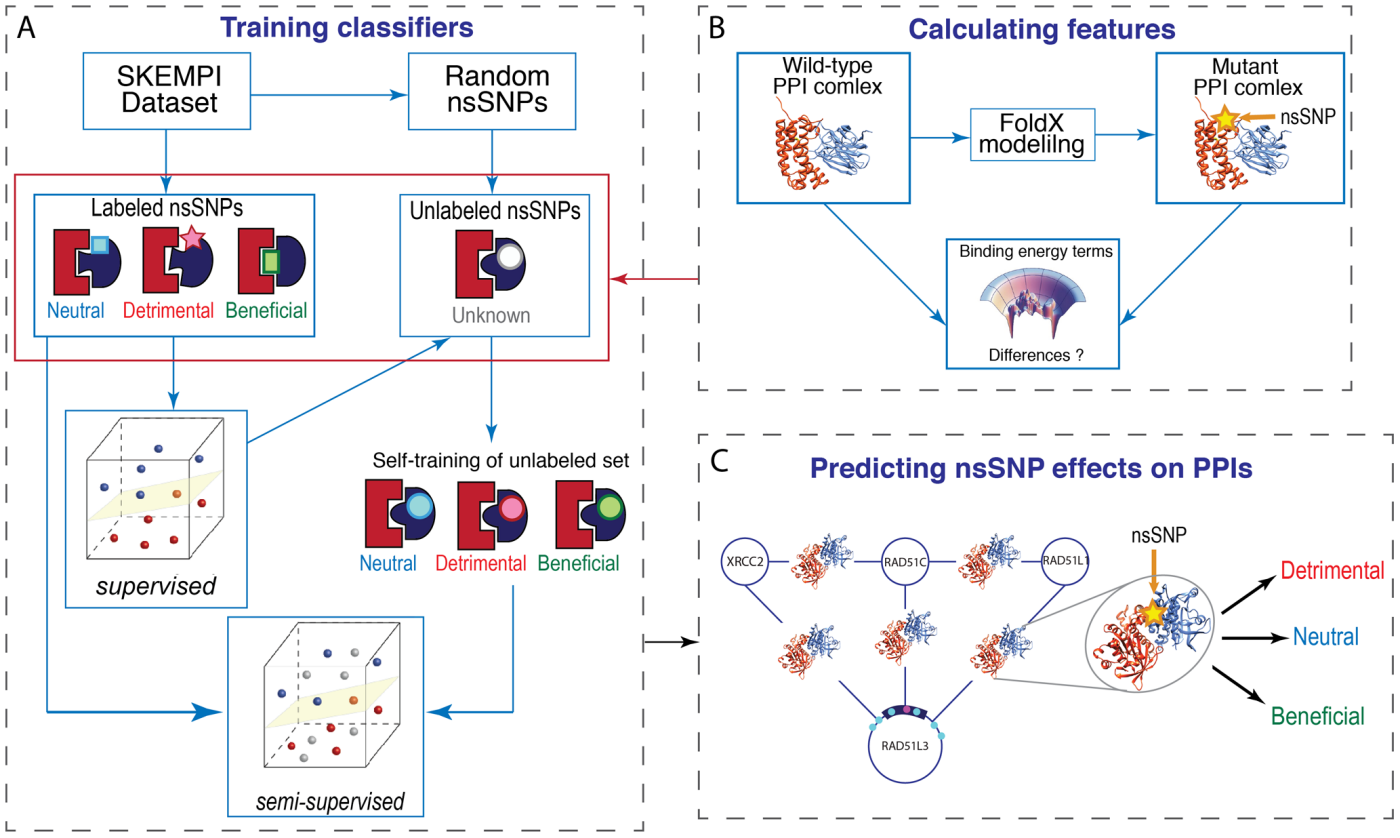
A flowchart of supervised and semi-supervised learning methods used to predict the effect of nsSNPs on PPIs. A. Shown is the protocol of training both supervised and semi-supervised methods for the 3-class problem (mutations of detrimental/neutral/beneficial effects). The semi-supervised learning method depicted here is the random-forest self-learning classifier. B. Feature representation of each nsSNP was calculated by taking energy differences between the wild-type and mutant complexes. The mutant PPI complex was modeled by FoldX using as a template the structure of wild-type complex. C. During the prediction stage, the classifier assigns a new nsSNP to one of the classes.

For each problem, supervised and semi-supervised approaches are developed and assessed, and their performances are compared. The top classifiers are then integrated into a computational tool called the SNP-IN (non-synonymous SNP INteraction effect predictor) tool. The overall protocol of the training stage includes four steps (Fig. 2). First, the data on nsSNPs are collected, and each nsSNP is assigned to a class by comparing the difference of binding affinity between the mutant and wild-type protein-protein interactions. Second, the unlabeled data are obtained by generating a complementary set of all other possible mutations different from the wild-type residue and its mutations analyzed as in the first step. These mutations are generated for each residue from the interaction interface of the PPI being analyzed. Third, for each nsSNP, a feature vector is generated. Last, a set of supervised and semi-supervised classifiers are trained and evaluated; for each classification problem a single classifier is selected. During the prediction stage, the same set of features for a novel nsSNP is calculated, and the feature vector is used to classify the nsSNP.

### Data collection and definition of interaction-associated types of nsSNPs

Comprehensive analysis of the mutation effects on PPIs on a large scale by experiments is a difficult task. As a result, while several datasets have been used by the computational methods [34]–[36], no golden standard currently exists. Here, we use one of the largest such datasets, SKEMPI [35], which includes mutations on structurally-defined heterodimeric complexes that were experimentally characterized and extracted and manually curated from the literature. For each mutation, the database provides the changes in thermodynamic parameters and kinetic rate constants between the wild-type and mutant PPIs. From the initially collected set of 3,047 mutations occurring in 158 heterodimeric complexes, we keep 2,795 mutations after removing the redundancy, where the redundant mutations are defined as the same mutations obtained from different references. Finally, since in this work we focus on the effects caused by a single nsSNP, we filter out from the sets those entries that include multiple mutations, resulting in the final dataset of 2,079 single SNPs and 151 corresponding protein complexes (This training dataset is available for download at SNP-IN tool website: http://korkinlab.org/snpintool).

Next, each mutation is characterized as one of three interaction-associated types: beneficial, neutral, or detrimental. The types are assigned based on the difference, 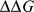, between the binding free energies of the mutant and wild-type complexes. Specifically, we calculate 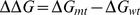, where 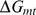 and 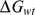 are the mutant and wild-type binding free energies, correspondingly. Each energy value is calculated as 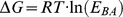, where 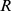 is the gas constant, 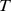 is temperature, and 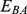 is the known binding affinity. For our dataset, 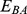 is obtained from the SKEMPI dataset at http://life.bsc.es/pid/mutation_database/datatable.html (column 7 for the mutant and column 8 for the wild-type). This value can also be calculated by 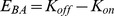, where 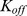, 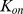 can also be found at the same link above. The beneficial, neutral, or detrimental types of mutations are then determined by applying two previously established thresholds to 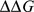 [35], [37], [38]:
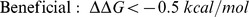


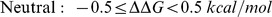


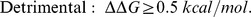



Intuitively, a neutral mutation will not change the interaction's properties, whereas the beneficial mutation will significantly increase the binding affinity, and the detrimental mutation is expected to disrupt the associated PPI. Using these three mutation types, the labeled dataset for each supervised and semi-supervised classifier is formed (see subsection *Training and evaluation of supervised and semi-supervised classifiers* in *Methods*). We note that these mutation types are introduced to characterize the effect on a protein-protein interaction rather than the biological function associated with the interaction. For instance, an nsSNP that has a beneficial effect on protein-protein interaction may have a detrimental functional effect by transforming a transient complex to a permanent one.

Finally, the dataset of unlabeled mutations is generated for the semi-supervised learning classifiers. Specifically, for each of the 2,079 mutations, all other 18 possible mutations, excluding the original mutant and wild-type residues, are introduced at the same location in the corresponding complex as the original nsSNP. For these mutations, no 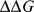 values are available, thus they cannot be assigned a specific interaction-associated type. The final set includes 17,692 mutations (mutations for which some of the software packages failed to generate the features are excluded).

### Feature representation

Each nsSNP in the labeled and unlabeled sets is represented as a 33-dimensional feature vector. To calculate the set of features, we first model the structure of the mutant PPI complex using FoldX [39], [40] and using the structure of the wild-type complex as a modeling template. Next, for each nsSNP a set of features is calculated for the modeled mutant complex as well as the wild-type native structure, and the difference of these features is included into the final feature vector.

Several software packages are used to generate the features (Table 1) [39], [41]–[45]. The first group consists of 22 energy terms calculated in FoldX: Total energy, Backbone Hbond, Sidechain Hbond, Van der Waals, Electrostatics, Solvation Polar, Solvation Hydrophobic, Van der Waals clashes, entropy sidechain, entropy mainchain, sloop_entropy, mloop_entropy, cis_bond, torsional clash, backbone clash, helix dipole, water bridge, disulfide, electrostatic kon, partial covalent bonds, Energy Ionisation, Entropy Complex [39]. The second group of three features includes energy terms (OPUS-PSP terms 1–3) calculated in OPUS-PSP [44]. Accessible surface area of the mutant amino acid residue is computed by NACCASS [41], as a descriptor to measure the changes on solvent accessibility during this mutation. The next feature, Interaction energy, is defined as the sum of interaction energies of the protein chain carrying the mutation against all other chains in the complex. Interaction energy for each pair of chains is also calculated in FoldX. The remaining features include three energy terms (Goap terms 1–3) from software Goap [45], Geometric score from Geometric tool [42], energy term from Dfire2 [46], and Decomplex energy score [43].

**Table 1:** Feature descriptions.

# Dimensions	Features	Descriptions
22	FoldX energy terms	22 energy values from FoldX output
3	OPUS terms	3 terms from OPUS
1	ASA	Naccess ASA of mutated residue
1	Interaction energy	FoldX interaction energy of mutated residue
3	Goap terms	3 terms from Goap
1	Geometric score	Energy score from Geometric
1	Dfire2 term	Energy from Dfire2
1	Dcomplex term	Energy score from Dcomplex

A 33-dimensional feature vector calculated for each nsSNP in both labeled and unlabeled sets. Each feature represents the difference in values of the corresponding terms calculated for the wild-type and mutant PPI complexes.

### Training and evaluation of supervised and semi-supervised classifiers

Two supervised and two semi-supervised approaches are implemented and compared. The supervised learning methods include Support Vector Machines (SVM) and Random Forrest (RF) classifiers, which have been consistently among the top performing methods for a number of bioinformatics tasks [47]–[49]. Random Forests have been shown to outperform other feature-based supervised learning approaches in bioinformatics and other domains [50]–[53], although in some cases they perform worse than SVM methods [48], [54]. The SVM approach, in addition to being among most widely used supervised learning methods in bioinformatics, lies in the core of the top performing semi-supervised learning algorithm [55]. For SVM, we assessed three popular kernels: (i) linear, (ii) polynomial kernel, 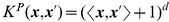, where *d* is degree of the polynomial, and (iii) radial basis function (RBF), 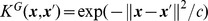. The polynomial kernel is then selected with *d* = 3 as the most accurate one, as it has the highest f-measure value. SVM models are implemented using the libSVM package [56] and the RF classifier is implemented in Weka software [57].

Semi-supervised learning has been only recently introduced to the field of bioinformatics [49], [58]–[61]. The basic idea is to rely not only on the labeled training data, but also to incorporate an additional, unlabeled, dataset (often of a significantly larger size) as a part of training to improve learning accuracy. We first apply semi-supervised learning by low density separation (LDS) [55], which is considered one of the most accurate semi-supervised methods [62]. The LDS approach relies on clustering to guide the unlabeled dataset by combining (i) graph-based distances that emphasize low density regions between clusters and (ii) optimization of the Transductive SVM objective function [63] which places the decision boundary in low density regions using gradient descent. Specifically, a nearest-neighbor graph *G* = (*V*,*E*) is first derived for both labeled and unlabeled feature vectors. Then a modified connectivity kernel 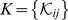 is computed, defined as follows:
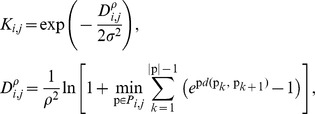
where p is a path of length |p| from the set P*_i,j_* of all paths connecting two feature vectors *x_i_* and *x_j_*, and 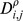 is a parameterized *ρ*-path distance defined between the set of all labeled vectors on one hand and set of all vectors on the other hand. The computed kernel is then used to train an SVM in the supervised part of the algorithm [55].

Based on assessment of the supervised methods (see *Leave-one-out cross validations* subsection), the RF classifier shows superior performance over the SVM classifiers. Thus, we would like to further improve the accuracy of this approach, by developing a simple RF-based semi-supervised learning protocol that leverages self-learning heuristics [64]. First the protocol trains a supervised learning RF classifier. Next, this classifier is applied to the unlabeled dataset and assigns each unlabeled nsSNP to one of the classes. The newly labeled dataset is merged with the originally labeled datasets. Finally, the resulting labeled datasets are used to re-train the supervised RF method. We note that while several RF-based semi-supervised based methods have been recently introduced in pattern recognition and computer vision [65], [66], to the best of our knowledge, no RF-based semi-supervised method has been applied in a bioinformatics area.

Finally, to further improve the performance on the most difficult 3-class problem, we explore whether the classifier of the 3–class problem can benefit from the other two classifiers addressing one of the 2-class problems. Specifically, for the most accurate classifier of Problem 3 (selected based on the weighted f-measure), we calculate two additional features: the prediction results from the most accurate binary classifiers for Problems 1 and 2. To obtain these features, we use each of the two binary classifiers to generate the prediction value if it is a positive prediction, or one minus prediction value if it is a negative prediction and scale the value to be from 0 to 1.

The labeled set for a supervised classifier addressing the first 2-class problem includes mutations determined as beneficial as the first class (strengthening PPI) and mutations determined as detrimental as the second class (weakening PPI). Another labeled set corresponding to the second 2-class problem includes both beneficial and neutral mutations as the first class (preserving PPI), and detrimental mutations as the second class (disrupting PPI). Mutations in the final labeled set corresponding to the 3-class problem are naturally grouped into beneficial, neutral, and detrimental classes. For each semi-supervised classifier, we use the same labeled data as in the corresponding supervised classifier and the previously described unlabeled set of 17,692 nsSNPs (Table 2).

**Table 2:** Training datasets for different classifiers.

	Problem 1	Problem 2	Problem 3
Beneficial	208	878 = 208(B)+670(N)	208
Neutral	-		670
Detrimental	1,076	1,076	1,076
Unlabeled	17,692	17,692	17,692

Different combinations of three types of nsSNPs are used for each of the three classification problems. The set of 878 PPI preserving mutations included both beneficial (B) and neutral (N) nsSNPs. The unlabeled set was used solely for semi-supervised learning methods.

To evaluate all supervised and semi-supervised classifiers for each of the three classification problems, three assessment protocols were implemented. The first protocol was a standard leave-one-out (LOO) cross-validation protocol with the goal to compare the methods and select the most accurate classifier for each problem by utilizing each of the labeled datasets for the corresponding problem in both supervised and semi-supervised cases. For each problem, the class-based recall, precision and f-measures are calculated for each class. Next, overall performance of a classifier on the classification problem is assessed by the average accuracy and weighted f-measure scores as following:
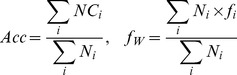
where *NC_i_*, *f_i_*, and *N*_i_ are the number of correctly identified class members, standard f-measure, and total number of class members in class *i*, correspondingly. A classifier with the highest weighted f-measure is selected for each problem and included into the SNP-IN tool web-server.

In the second protocol, we compare our top performing classifier with the only other published method for predicting the effect of nsSNPs on PPIs, BeAtMuSiC [31]. Unlike our approach, BeAtMuSiC relies on a set of statistical potentials derived from the structures of interacting proteins and does not use a supervised learning and, subsequently, a training set. Coincidentally, for the assessment of this method the authors used the same SKEMPI dataset as was used in SNP-IN tool LOO cross-validation, with a slightly different redundancy removal protocol. Thus, we compared the performances of BeAtMuSiC and SNP-IN tool on the overlapped dataset by calculating the Pearson correlation coefficient between the predicted scores and the experimental data for the latter predictor and comparing with the published score for the former method. The raw classification prediction score of the SNP-IN tool was used. We discuss the validity and potential shortcomings of this assessment protocol further in the paper.

In the last protocol, we assess the performance of SNP-IN tool by applying it to the datasets of 26^th^ Critical Assessment of PRediction of Interactions (CAPRI) competition [67]. CAPRI is a community-wide competition in computational tasks related to characterization of the molecular structure of protein complexes. Recently, a new type of challenge was introduced with a goal to characterize the effect of mutation on protein-protein complexes. Specifically, there were two challenge targets (Target 55 and Target 56), each target was a designed influenza inhibitor interacting with hemagglutinin (HA) [68]. A comprehensive set of site-directed mutagenesis experiments was done for the residues located next to or inside the interaction interface for each target complex, and the effect of each point mutation on the binding affinity was evaluated by deep sequencing of mutants before and after binding [69]. During the competition, all CAPRI participants were asked to provide a score as the prediction of each mutation's effect on inhibitor-HA interactions. The three types of effects correspond to our 3-class problem and include detrimental, neutral and beneficial mutations. The correlations between predicted scores and experimental evaluations were calculated by using the Kendall's *τ* rank correlation coefficient (http://www.ebi.ac.uk/msd-srv/capri/round26/). Here, we apply the CAPRI assessment protocol to predictions of the effect of each point mutation in Targets 55 and 56 obtained by the 3-class classifier from SNP-IN tool.

### Case study protocol

Finally, the SNP-IN tool is applied to analyze nsSNPs in the PPI networks associated with human diseases in two case studies using the following protocol. First, the disease-associated nsSNPs and the corresponding genes are selected from dbSNP database [70]. Second, for each nsSNP, a PPI mediated by the mutated protein is identified, and its structural template is extracted from a recently published dataset by Wang *et al* [7]. Third, MODELLER [71] is used to build an accurate comparative model for each nsSNP-associated PPI complex. Last, SNP-In tool is used to predict nsSNP-induced loss/preservation of the PPI by characterizing the effect of that nsSNP on the PPI.

### Web-server

**Figure 3:**
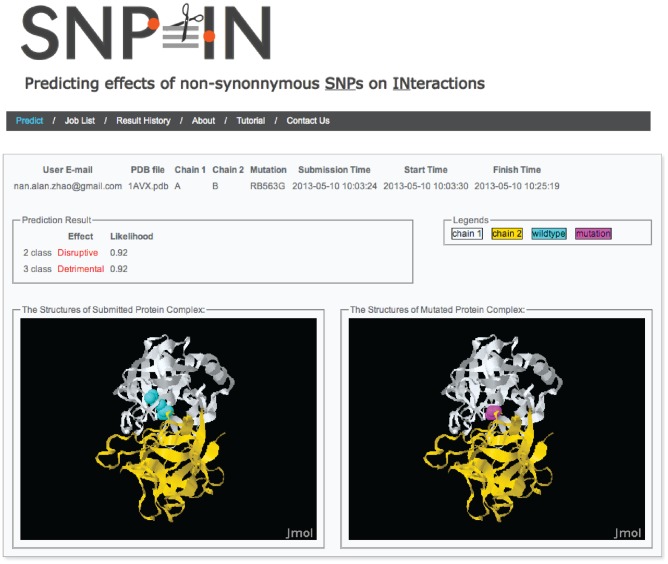
Snapshots of SNPIN-tool server. Snapshots of the result visualization page of SNPIN-tool web server, freely available at http://korkinlab.org/snpintool/. The submission page allows users to upload a pdb file of the PPI structure and specify interacting chains with a mutation (nsSNP). When the submitted job is finished, SNPIN-tool returns the prediction results and the estimated likelihoods for the 2-class and 3-class classifiers. In addition, 3D structures of both wild-type and mutant PPIs with the highlighted residue position where nsSNP occurred are visualized.

The SNP-IN tool was implemented as a web-server freely available at http://korkinlab.org/snpintool/ (Fig. 3). It allows users to upload a pdb file containing the structure of the studied PPI, and provide information about the nsSNP they would like to investigate. The server will then return the effects of the nsSNP predicted by the semi-supervised RF-SL classifiers for both 2- and 3-class problems.

## Results

Here, we provide a comparative assessment of the supervised and semi-supervised approaches with (i) each other, (ii) the only currently published method, and (iii) the results of a recent CAPRI competition. We also analyze the importance of contribution of each feature in each of the three classification problems. Finally, we report results of the application of SNP-IN tool to characterization of genomic variants in the PPI networks associated with two human diseases.

### Feature ranking

The importance analysis of all 33 features, carried out using InforGainAttributeEval function in Weka [72], showed that many features (Table 3) were equally important for all three classification problems. These are primarily the energy terms obtained from FoldX and OPUS. On the other hand, some features appeared to be important only for certain classification problems. For instance, Geometric score and Accessible Surface Area (ASA) were not important in the interaction disrupting/preserving classification problem, while the Goap energy terms were more important, compared with the other two problems. On the other hand, Electrostatics feature appeared to be more important for the 3-class problem than for the 2-class problems. Interestingly, while relative contribution of the features was different, all features without exception were informative in the vector representation: removing each of the features did not improve the prediction accuracy for any of the supervised methods. The importance analysis, thus, may be used to determine a higher priority when improving the accuracy of certain features, such as the FoldX and OPUS energy terms, which may be beneficial for all three classification problems.

**Table 3:** Feature importance ranking.

Ranking	Classification (1)	Classification (2)	Classification (3)
1	Interaction Energy	Interaction Energy	Interaction Energy
2	Dcomplex term	Total energy	Dcomplex term
3	Geometric score	OPUS term 1	Geometric score
4	Total energy	Backbone clash	Total energy
5	OPUS term 1	Dcomplex term	OPUS term 2
6	OPUS term 2	OPUS term 2	OPUS term 1
7	Vander Waals	Vander Waals	Vander Waals
8	Solvation Polar	Dfire2 term	Solvation Polar
9	Goap term 2	Solvation Hydrophobic	Electrostatics
10	Dfire2 term	Goap term 2	ASA
11	Side chain H-bond	Goap term 1	Goap term 2
12	Solvation Hydrophobic	Electrostatic kon	Dfire2 term
13	Vander Waals clashes	Entropy sidechain	Solvation Hydrophobic
14	ASA	Vander Waals clashes	Electrostatic kon
15	Backbone clash	Torsional clash	Vander Waals clashes
16	Electrostatic kon	Side chain H-bond	Goap term 1
17	Goap term 1	Solvation Polar	Backbone clash
18	OPUS term 3	Electrostatics	Torsional clash
19	Electrostatics	Entropy mainchain	Side chain H-bond
20	Torsional clash	Backbone H-bond	Backbone H-bond
21	Backbone H-bond	Geometric score	OPUS term 3
22	Entropy side chain	Helix dipole	Energy Ionisation
23	Entropy main chain	Energy Ionisation	Goap term 3
24	Energy Ionisation	Entropy Complex	Entropy mainchain
25	Helix dipole	Partial covalent bonds	Helix dipole
26	Partial covalent bonds	Goap term 3	Entropy sidechain
27	Disulfide	OPUS term 3	Disulfide
28	Goap term 3	ASA	Entropy Complex
29	Entropy Complex	mloop_entropy	Partial covalent bonds
30	mloop_entropy	sloop_entropy	mloop_entropy
31	sloop_entropy	Disulfide	sloop_entropy
32	Water bridge	cis_bond	Water bridge
33	cis_bond	Water bridge	cis_bond

Feature importance rankings calculated using InforGainAttributeEval function in Weka for each of the three classification problems.

### Leave-one-out cross validations

To assess performance of the four classifiers, we applied a LOO cross-validation protocol (Table 4, Table S1). We started by testing the classifiers on the data for the first classification problem (strengthening/weakening mutations). Interestingly, for all four classifiers, predicting a weakening mutation was significantly more accurate than predicting a strengthening one. In addition, both the SVM supervised classifier and LDS semi-supervised classifier, which relied on transductive SVM (TSVM), performed worse than the RF-based supervised and RF-based semi-supervised learning methods. The top performing RF-based supervised classifier reached 0.87 in weighted f-measure and 0.89 in average accuracy.

**Table 4:** Leave-one-out cross-validation results.

		Classifier	Classes	Recall	Precision	f-measure	*f_W_*	*Acc*
Problem 1	Supervised	SVM	Strengthening	0.24	0.67	0.35	0.83	0.86
			Weakening	0.98	0.87	0.92		
		**RF**	Strengthening	0.37	0.88	0.52	**0.87**	**0.89**
			Weakening	0.99	0.89	0.94		
	Semi-Supervised	LDS- TSVM	Strengthening	0.40	0.42	0.41	0.81	0.81
			Weakening	0.89	0.89	0.89		
		RF-SL	Strengthening	0.32	0.94	0.48	0.86	0.89
			Weakening	1.00	0.88	0.94		
Problem 2	Supervised	RF	Preserving	0.69	0.72	0.70	0.74	0.74
			Disruptive	0.78	0.75	0.77		
	Semi-supervised	**RF-SL**	Preserving	0.71	0.78	0.74	**0.78**	**0.78**
			Disruptive	0.83	0.78	0.80		
Problem 3	Supervised	SVM	Beneficial	0.00	0.00	0.00	0.50	0.59
			Detrimental	0.96	0.58	0.73		
			Neutral	0.19	0.67	0.29		
		RF	Beneficial	0.28	0.78	0.41	0.68	0.70
			Detrimental	0.85	0.71	0.78		
			Neutral	0.58	0.66	0.62		
	Semi-supervised	LDS-TSVM	Beneficial	0.07	0.13	0.09	0.41	0.41
			Detrimental	0.48	0.53	0.51		
			Neutral	0.40	0.31	0.35		
		RF-SL	Beneficial	0.22	0.80	0.34	0.70	0.72
			Detrimental	0.89	0.71	0.79		
			Neutral	0.60	0.73	0.66		
		**RF-SL-2F**	Beneficial	0.40	0.86	0.55	**0.75**	**0.78**
			Detrimental	0.91	0.78	0.84		
			Neutral	0.69	0.78	0.73		

Recall, precision, and f-measure are calculated for each class. Weighted f-measure, *f_W_*, and average accuracy, *Acc*, are calculated for all classes of a problem. All assessments are based on leave-one-out cross-validation on the labeled dataset. Shown in bold are the top-performing classifiers for each problem. RF-SL-2F corresponds to the self-learning RF classifier using 2 additional features.

The performance gap between the SVM-based and RF-based methods became even more apparent when assessing these methods on the 3-class problem (Problem 3). Specifically, very low recall and precision when classifying the beneficial nsSNPs made the difference between the weighted f-measures of SVM-based and RF-based methods to be close to 0.20 for both supervised and semi-supervised approaches (Table 4). The top performing method for this classification problem was the RF-based semi-supervised approach, with the weighted f-measure value of 0.70 and average accuracy of 0.72.

Based on the superior performance of the supervised and semi-supervised RF-based methods for the first 2-class and 3-class problems, we focused on evaluating only those two methods for the second 2-class problem (disruptive/preserving PPI mutations). We found that unlike the previous two classification problems, the performance of both methods on the two classes of this problem was more even (Table 4). Interestingly, the top performing RF-based semi-supervised approach for this problem (weighted f-measure is 0.78 and average accuracy is also 0.78) gained ∼0.04 in weighted f-measure, compared to the supervised approach. This was not observed in the other two classification problems where the difference between the RF-based supervised and semi-supervised classifiers was at most 0.02.

The results of cross-validation allowed us to select the top performing method for each problem, using weighted f-measure (Table 4). The top classifiers for the more generally applicable second and third classification problems were then integrated into the SNP-IN tool. The overall weighted prediction accuracies (0.72–0.89) and f-measures (0.70–0.87), as estimated by the LOO cross-validation protocol, suggest that each of the three problems is feasible when applying a machine learning approach. In addition, we observed that the performance of the classifiers on individual classes varies even in the case of the most accurate methods. To account for that in our evaluation, we calculated the Mathews correlation coefficient (MCC) score for the top-performing RF approaches (Table S1). The overall performance of the methods according to the MCC score was consistent with the performance evaluated based on the weighted f-measure.

While the thresholds for 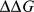 employed here are widely used by the community [35], [37], [38], other more conservative definitions for the beneficial/neutral/detrimental mutations exist. For instance, Bogan and Thorn [73] used a threshold of 2.0 kcal/mol to identify the residues that contributed to the interaction hot spots. We analyzed and compared the behavior of our top performing supervised and semi-supervised methods by defining beneficial, neutral, and detrimental effects using the more conservative thresholds of ±2.0 kcal/mol instead of ±0.5 kcal/mol, followed by retraining and evaluation of the methods for each problem (Table S2). Using the more conservative definition resulted in significantly unbalanced datasets (beneficial: 48, neutral: 1388, detrimental: 518), but the performance of the classifiers was similar, showing that our approach is adaptive to other definitions of interaction effects.

Lastly, by including the performance of the two 2-class classifiers as additional two features we were able to get a striking improvement of the most accurate RF self- learning classifier for the 3-class problem (Table 4, last row). Most significantly, we obtained 82% gain in the recall of classifying beneficial mutations (from 0.22 to 0.40), and 25% gain of the MCC score (from 0.49 to 0.61). Thus, integrating the intrinsic relationship between classification problems allowed us to significantly improve predictions for the most difficult 3-class problem. We note that there may be other, simpler, 2-level protocols where each of the three classes can be eliminated consecutively (*e.g.*, classifying the detrimental nsSNPs *vs.* the rest at the first level, and classifying the neutral nsSNPs *vs.* beneficial ones at the second level). However, our protocol is less restrictive, since it does not make a classification decision for all three classes until the last level, where the performances of both 2-class classifiers are considered simply as additional numerical features and may or may not influence the final classification.

### Comparison to BeAtMuSiC on SKEMPI set

We next compared the performance of our top performing RF-based semi-supervised classifier to BeAtMuSiC, a recently published and the only publicly available tool, to the best of our knowledge [31]. The authors of BeAtMuSiC assessed their method by applying it to the SKEMPI set. Out of 3,047 entries in SKEMPI, they removed the redundant entries and entries with multiple mutations. The resulting set of 2,007 was used to calculate the predicted values and compare them with the original experimental measurements. Following our preprocessing protocol, we also removed redundant entries and entries with multiple mutations and then successfully predicted 1,954 mutations. Finally, comparing our set with the set of 2,007 entries used in BeAtMuSiC, we determined 1,897 entries shared between the two sets that we used for our comparative assessment.

We note that BeAtMuSiC is not a classifier, as it predicts the changes in binding affinity caused by an nsSNP. Therefore, instead of direct classification results, we used the classifier-calculated probability for an nsSNP to be of the preserving type; we expected this probability to correlate well with changes in the binding affinity. We also note that our RF-based classifier and all other classifiers were trained using the SKEMPI set. Therefore, for this comparative assessment we applied a LOO cross-validation protocol to train models and used predictions on the test examples from the same protocol to calculate the Pearson correlation coefficient [31]. As a result, the computed Pearson correlation coefficient between our prediction scores and experimental values from SKEMPI was 0.57, while the authors of BeAtMuSiC reported the correlation coefficient of 0.47.

### Validation on the dataset from the 26th round of Critical Assessment of PRediction of Interactions (CAPRI)

As a final evaluation of our method, we applied the semi-supervised RF-SL classifier of SNP-IN tool to characterize all mutations of both CAPRI Targets, 55 and 56, and then scaled the probability of each classification to obtain the score of mutation effects on binding. Comparing to other participation groups in 26^th^ round of CAPRI [74] and BeAtMuSiC applied for the same purpose [31], our RF-SL classifier from SNP-IN tool obtained a Kendall's tau coefficient with experimental results of 0.37 on target 55 and 0.25 on target 56. Both results were significantly better than those ones by either a CAPRI predictor or BeAtMuSiC (Table 5). The validation on the targets of the 26th round of CAPRI demonstrates that our semi-supervised RF-SL classifier is currently the best predictor of the mutation effects on PPIs.

**Table 5:** Kendall's tau rank correlation coefficient on target 55 and target 56 of the 26-th round of CAPRI.

Predictors	Kendall's tau rank correlation coefficient	
	Target 55	Target 56
SNP-IN tool	**0.369**	**0.249**
BeAtMuSiC	0.290	0.190
CAPRI Group 1	0.150	0.019
CAPRI Group 2	0.061	0.056
CAPRI Group 3	0.080	0.035
CAPRI Group 4	0.098	0.029
CAPRI Group 5	0.094	0.085
CAPRI Group 6	0.141	0.079
CAPRI Group 7	0.077	0.041
CAPRI Group 8	0.066	0.129
CAPRI Group 9	0.163	0.044
CAPRI Group 10	0.224	0.214
CAPRI Group 11	0.166	0.139
CAPRI Group 12	0.039	0.077
CAPRI Group 13	0.088	0.016
CAPRI Group 14	0.295	0.172
CAPRI Group 15	0.286	-
CAPRI Group 16	0.165	0.147
CAPRI Group 17	0.123	0.054
CAPRI Group 18	0.054	0.015
CAPRI Group 19	0.131	−0.029
CAPRI Group 20	0.134	0.173
CAPRI Group 21	0.068	0.047
CAPRI Group 22	0.232	-

Kendall's tau coefficients between predicted scores and experimental evaluations were calculated for our semi-supervised RF-SL classifier of SNP-IN tool, a recently published predictor (BeAtMuSiC [31]), and all groups participating in the 26^th^ round of CAPRI competition (http://www.ebi.ac.uk/msd-srv/capri/round26/).

### Application: Studying the nsSNP-induced rewiring of disease interaction networks

The accuracy and computational performance of our approach allowed us to study the mutation-induced rewiring effects of protein-protein interaction networks mediated by disease genes. The rationale of this approach was as follows. All nsSNPs on the surface of a protein could be roughly organized in two groups with respect to their role in a PPI mediated by this protein. The first group included nsSNPs that were located inside the interaction interface, while the second group consisted of nsSNPs that are located outside interface (but might nevertheless rewire the PPI).

**Figure 4:**
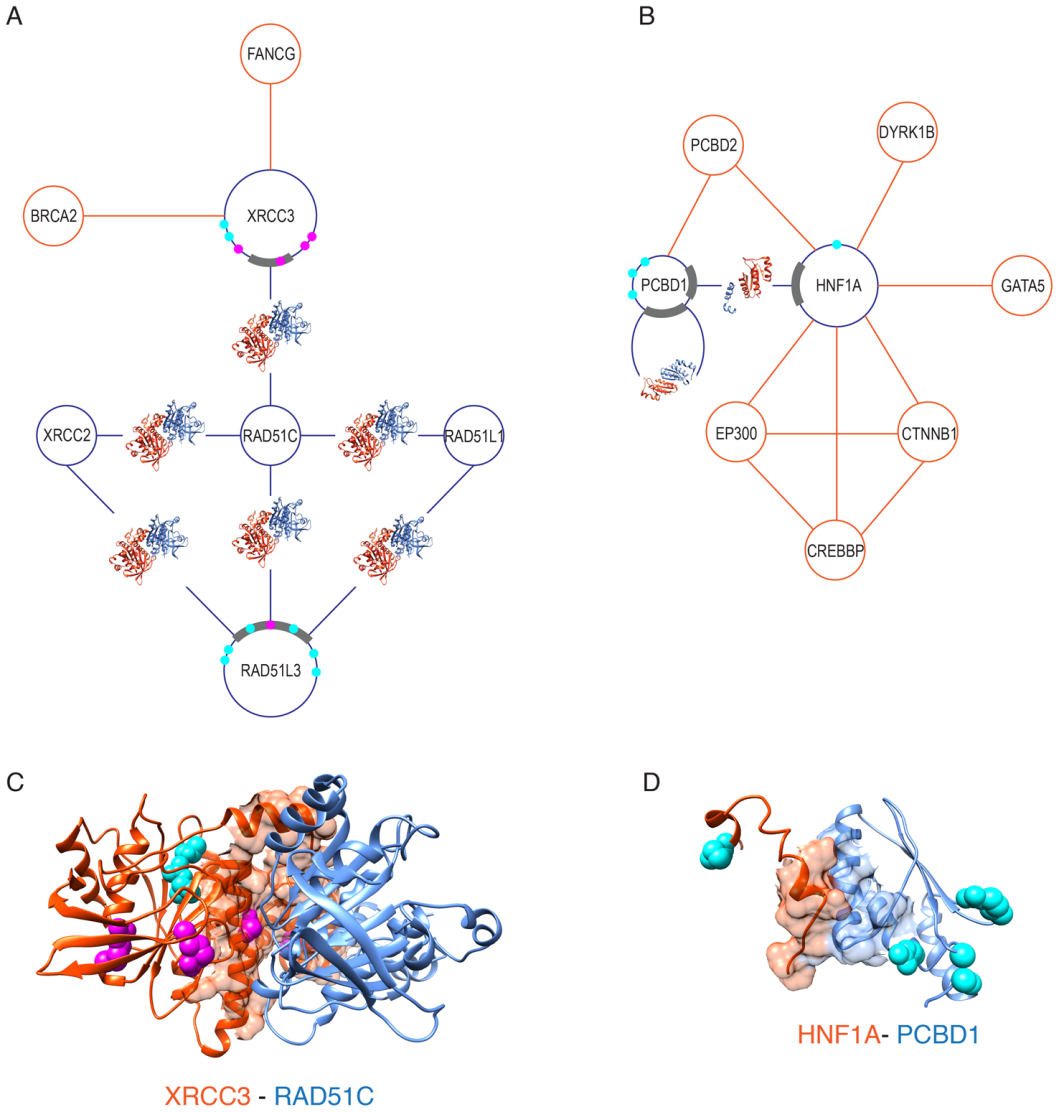
Effects of disease-associated nsSNPs rewiring disease interaction networks. SNP-IN tool is applied to study two disease-centered PPI networks. Each PPI network consists of several binary interactions, some of which are covered by structural templates (shown as protein structures inside the network edges). Binding sites of the corresponding interaction interfaces are shown in grey. The results of nsSNP classification by SNP-IN tool into disruptive (magenta) and preserving (cyan) are shown for proteins whose interactions are structurally covered. A. A sub-network of seven breast cancer associated genes. B. A sub-network of eight diabetes associated genes. C. A structure model of PPI between XRCC3 and RAD51C interacting domains with predicted nsSNP effects. D. A structure model of PPI between HNF1A and PCBD1 interacting domains with predicted nsSNP effects.

To demonstrate the applicability of our approach, we used it to study two disease PPI networks centered around the genes critically implicated in two complex genetic diseases, breast cancer and diabetes (Fig. 4). For each study, we used dbSNP [70] and a recently published INstruct database [75] to (1) select the disease-associated genes that form a PPI network, (2) select nsSNPs associated with the disease, and (3) determine whether any interactions from that network have homologous structural templates. To ensure the accuracy of the PPI data we used HINT database [76] that includes PPIs experimentally supported by one or more publications. We required for each PPI to be supported by at least two references. For each PPI with a known structural template we obtained a homology model (see Feature representation subsection in Methods), mapped known nsSNPs onto the modeled structure of the PPI and grouped them into the two groups discussed above. Finally, we run SNP-IN tool on each structurally resolved PPI and compared the obtained results with the known literature on the effects of those variants.

#### Case Study 1: PPI network of breast cancer associated genes

We extracted seven genes, XRCC3, XRCC2, RAD51C, RAD51L1, RAD51L3, FANCG, BRCA2, that formed a connected PPI network with eight interactions (Fig. 4 A), and had been associated with breast cancer as well as other types [77], [78]. The three most connected proteins, RAD51C, RAD51L3, and XRCC3 had been critically implicated in the combinatorial DNA repair due to the damage from ionizing radiation, mutagenic chemicals and other DNA-damaging agents; the genetic variants of these genes had been directly or indirectly linked to the disease [79]–[81]. Specifically, forming an interaction complex between RAD51C and XRCC3 was found to facilitate DNA-binding [82]. On the other hand, RAD51C and RAD51L3 were found to be a part of a larger complex with the proposed function of forming filaments on ssDNA, necessary for the formation of paired DNA molecules and subsequent strand exchange and recombination [78], [83].

Six of eight PPIs had at least one structural template covering them. Interestingly, all six interactions were mediated by paralogous pairs of domains and thus could be modeled using the same template (PDB ID: 1PZN). Two proteins, RAD51L3 and XRCC3, had six and seven diseases-associated nsSNPs, correspondingly (Table S3), covered by PPI structural templates. The first template covered RAD51L3-RAD51C interaction and therefore was used to model it, while the second template was used to model RAD51L3-RAD51C. When we applied SNP-IN tool to characterize each of the nsSNPs, we found that the majority of the disruptive mutations were located on XRCC3 (four out of six disruptive nsSNPs, including one directly in the binding site (Fig. 4 C), while the majority of the nsSNPs on the surface of RAD51L3 (six out of seven nsSNPs, including two on the binding site) were predicted to be preserving the PPI (Fig. S1 A). Interestingly, all four of disruptive nsSNPs in XRCC3 are mutations of arginine (Fig. 4 C). We note that determining the disruptive effects of nsSNPs using SNP-IN tool may not be sensitive to the cases when these mutations trigger such mechanisms indirectly. For instance, recent functional analysis of E233G mutation in RAD51L3 found a two-fold decrease in the interaction of the protein with RAD51C, compared to the wild-type [84]. The authors suggested that the mutation residue might disrupt the inter-domain interactions RAD51L3, altering protein structure and folding of the protein, which in turn affected its interaction with RAD51C. As there is no evidence for the direct mechanism of rewiring the interaction by E233G, SNP-IN tool characterized as a neutral mutation. Since RAD51L3 was also found to interact with XRCC2 and RAD51L2, and both interactions were modeled (using the same template), effects of the same set of nsSNPs on those two interactions were also predicted by SNP-IN tool (Fig. S1 B and C). All nsSNPs from RAD51L3 were characterized as neutral for each of the two interactions (Table S3).

Many of the reported mutations are yet to be studied, however several genetic variants have been analyzed extensively including other cancer types. For instance, the mutation T241M of XRCC3 has been previously identified a potential contributor to breast cancer in one study, whereas no association with either breast or skin cancer was found in another study. The fact that this mutation occurs outside the interaction interface and the fact that it was predicted to be preserving by SNP-IN tool suggest that it does not have a direct impact on the PPI, which would drastically change the functioning of the interacting proteins. Our findings are in concordance with the recently proposed hypothesis that instead of a stronger genetic-only effect associated with this variation, gene-environment interactions are required, for which the environmental exposure may not be present in some study groups and which would explain the different outcomes of association studies [85].

#### Case Study 2: PPI network of type II diabetes mellitus associated genes

We found eight genes associated with several forms of diabetes [86], which formed a connected PPI network (Fig. 4 B). While, similar to the first case, each interaction was supported by at least two PubMed references, only two out of ten determined interactions had structural templates covering the interaction interface, HNF1A-PCBD1 and PCBD1 homodimer. Those two interactions are intrinsically related with each other: PCBD1 (also referred to as DCoH), being a dimerization co-factor of HNF1A, binds its dimer domain [87]. Both proteins are co-expressed in liver, kidney, small intestine, and pancreas tissues and are implicated in the enzymatic activity. None of three variants of PCBD1 or a variant of HNF1A covered by a structural template (Table S3) was found in the interaction interface of either interaction. SNP-IN tool predicted all four variants to have a preserving effect on the two PPIs (Fig. 4 D and Fig. S1 D). Unlike the first case, we could not find any published evidence that any of these nsSNPs are causative mutations. Interestingly, a recent report associated I27L of HNFA1 with a “protective” effect to hypertriglyceridemia [87]. With the new structural templates available, it will be possible characterize nsSNPs associated with other genes in the diabetes-centered PPI network.

## Discussion

In this work, we developed a new approach, SNP-IN tool, that characterizes the effects of nsSNPs on protein-protein interactions. We introduced three related nsSNP effect classification problems and applied supervised and semi-supervised machine learning methods leveraging SVM and RF formalisms. The performance assessment of the classifiers allowed us to draw several conclusions regarding the nature of the studied problem and the machine learning methodology addressing it. First, we found that while many of the same nsSNP features play equally important role in all three classification problems, some problems appeared to be more challenging than the others. Second, we concluded that the random forest approach is better suited for this problem than the SVM approach: both RF-based supervised and semi-supervised methods significantly outperformed the corresponding SVM-based methods. Finally, we observed that the semi-supervised learning method did not always significantly outperform the supervised method. The comparative assessment showed the superior performance of SNP-IN tool on the CAPRI targets as well as over the only other published method, BeAtMuSiC. We note, however, that the latter comparison should be treated with caution, as it was done over the SKEMPI dataset that was used in LOO for SNP-IN tool. In contrast, BeAtMuSiC is not a machine learning approach, so it used this dataset exclusively for its assessment. Thus, while none of the assessed examples from SKEMPI were simultaneously used in training (due to design of LOO cross-validation protocol) and could not influence the classifiers, further more detailed assessment between these two methods must be done, when another large dataset is available.

Semi-supervised learning approaches have received growing attention from the bioinformatics community with their successful applications to several areas of bioinformatics and computational biology [47]–[49]. To the best of our knowledge, none of the currently existing semi-supervised approaches in bioinformatics have utilized random forest classifiers. Our simple RF-based semi-supervised classifier performed remarkably better than state-of-the-art transductive SVM and LDS based semi-supervised classifiers, suggesting that this could be a promising direction for addressing the biological classification problems that involve vector-based representations of highly heterogeneous features. Overall, limitation of the labeled data due to the difficulty of obtaining experimental binding affinities from the site-directed mutagenesis experiments renders semi-supervised approaches a powerful alternative to the supervised methods.

A related issue is predicting the effect of a non-synonymous SNP on a function carried by a protein product of the mutant gene, and specifically on a PPI mediated by this protein, has emerged as an important computational challenge. A problem of labeling nsSNPs as detrimental, neutral or beneficial, has been recently introduced for the first time at the 26th round of the CAPRI competition [88]. Considering the 3-class problem as the most comprehensive annotation for nsSNP effects on PPI, we have also introduced two other problems, each involving only 2 classes. While related, the problems are designed to characterize the genetic variation from different perspectives. One two-class problem, where an nsSNP is characterized as disrupting or preserving the associated PPI could be used to study the network rewiring caused by certain mutations, which in turn could be useful in pinpointing the causative SNPs. The other 2-class problem, where an nsSNP is labeled as either strengthening or weakening the interaction, is useful when characterizing molecular mechanisms behind a SNP that has been already linked to a functional change.

While an nsSNP occurring inside or in close proximity of an interaction interface will directly modify only one of the two interacting proteins, it is critical that our method takes into account the structural information of the entire interaction, including both binding sites forming the interaction interface. In this manner, the role of the interaction partner and its binding site is taken into consideration. For instance, it is possible that for a protein that competitively binds two other proteins through fully or partially overlapping binding sites, a mutation occurring in the overlapping region of these binding sites would disrupt one interaction but be neutral for another interaction. With hundreds of thousands of available interaction templates [89] and the advancement of comparative modeling, the requirement for structural information of the overall interaction makes an increasingly small impact on the coverage of SNP-IN tool.

Understanding functional roles of nsSNPs associated with diseases by studying the disease-centered PPI network has many challenges. Being among the first such methods, SNP-IN tool is yet to deal with some of them. One of the key challenges is accounting for the indirect effects of nsSNPs on the interactions, such as disabling a phosphorylation site that regulates a PPI, altering an allosteric site, or nsSNP-induced structural changes of a protein that affect the interaction. The difficulty of modeling such effects lies in the complexity of indirect mechanisms, as well as in the fact that the effect-causing SNPs may be relatively distant from the protein interaction interface they affect.

Another challenge is our ability to infer the functional importance of an nsSNP—and ultimately its contribution to the disease phenotype—from prediction of its effect on a PPI. For instance, the disruptive effect on a PPI predicted for an nsSNP that is either buried inside the interface or lies in its close proximity would indicate the true functional effect of the variation. However, predicting the neutral effect of a surface nsSNP that is in proximity to the interface does not necessarily mean that this genetic variation does not alter a biological function, as it could be a part of another functional site. On the other hand, an nsSNP that is buried inside the protein interaction interface is far less likely to be involved in the other function, *e.g.*, belong to a DNA- or small ligand–binding site or a site of posttranslational modification. Thus, the predicted neutral effect of such genetic variation would indeed mean that it does not have any functional impact.

As a recent work by Wang et al showed [7], there are thousands of nsSNPs associated with the interaction interfaces, and more SNPs are being identified every year from new high-throughput studies [90]. Combined with the exponential growth of the number of PPI structures being experimentally solved [91], we expect that the coverage of SNP-IN tool will continue to grow, providing more insights into molecular mechanisms of complex genetic diseases. In addition, with the growing experimental knowledge about the cooperative effects of multiple nsSNPs on PPIs, we plan to expand the SNP-IN tool to multiple mutations as one of the next future steps. Even more challenging is a problem of computational estimation of the 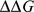 values upon structural changes in the protein interaction complex due to genetic variation. The classification of nsSNPs can be considered as a simplified, discretized, version of the latter problem. Based on the success of the current machine learning approach, we anticipate that the supervised and semi-supervised regression approaches will complement the classical biophysical methods to address this challenge.

## Supporting Information

Figure S1 **Structure models of disease associated PPIs with predicted effects of nsSNPs.** A. Structure model of PPI between RAD51L3 and RAD51C. B. Structure model of PPI between RAD51L3 and RAD51L1. C. Structure model of PPI between RAD51L3 and XRCC2. D. Structure model of PPI of a homodimer formed by PCBD1. Preserving nsSNPs are shown in cyan and disruptive ones are shown in magenta.
(TIF)

Table S1 **Mathews correlation coefficient (MCC) score for the top-performing RF approaches.** Different combinations of three types of nsSNPs are used for each of the three classification problems. RF, RF-SL, RF-SL-2F correspond to the supervised random forest classifier, self-learning random forest classifier, and self-learning random forest classifier using 2 additional features (predictions of effects by adding results from the 2-class classifiers), correspondingly.
(DOCX)

Table S2 **Leave-one-out cross validation results for the top performing supervised and semi-supervised methods trained using more conservative thresholds of ±2.0 kcal/mol.** Recall, precision, and f-measure are calculated for each class. Weighted f-measure, *f_W_*, average accuracy, *Acc*, and *MCC* score are calculated for all classes of a problem. All assessments are based on leave-one-out cross-validation on the labeled dataset.
(DOCX)

Table S3 **Disease associated mutations studied in the two case studies.** Predictions are made using the most accurate classifier for the second 2-class problem, disruptive (D) and preserving (P) PPI mutations.
(DOCX)
