# Supplementary figures and images for: Determining Effects of Non-synonymous SNPs on Protein-Protein Interactions using Supervised and Semi-supervised Learning
Source: PLoS Comput Biol. 2014 May 1;10(5):e1003592. doi: 10.1371/journal.pcbi.1003592 (PMC4006705; doi:10.1371/journal.pcbi.1003592)

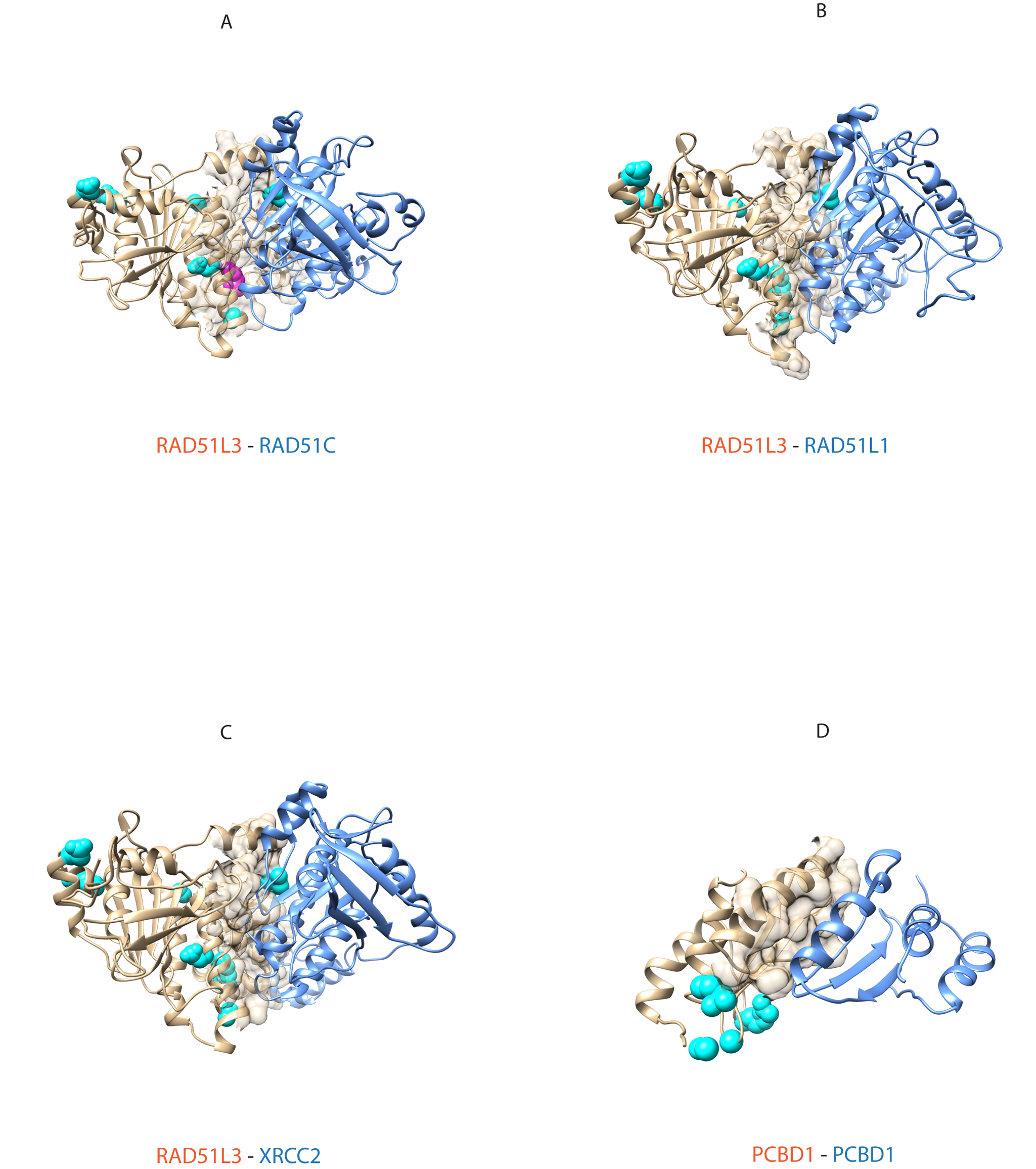

Supplement: Figure S1 — Structure models of disease associated PPIs with predicted effects of nsSNPs. A. Structure model of PPI between RAD51L3 and RAD51C. B. Structure model of PPI between RAD51L3 and RAD51L1. C. Structure model of PPI between RAD51L3 and XRCC2. D. Structure model of PPI of a homodimer formed by PCBD1. Preserving nsSNPs are shown in cyan and disruptive ones are shown in magenta. (TIF) [file pcbi.1003592.s001.tif]
