# Supplementary material for: Determining Effects of Non-synonymous SNPs on Protein-Protein Interactions using Supervised and Semi-supervised Learning
Source: PLoS Comput Biol. 2014 May 1;10(5):e1003592. doi: 10.1371/journal.pcbi.1003592 (PMC4006705; doi:10.1371/journal.pcbi.1003592)
Supplement: Table S1 — Mathews correlation coefficient (MCC) score for the top-performing RF approaches. Different combinations of three types of nsSNPs are used for each of the three classification problems. RF, RF-SL, RF-SL-2F correspond to the supervised random forest classifier, self-learning random forest classifier, and self-learning random forest classifier using 2 additional features (predictions of effects by adding results from the 2-class classifiers), correspondingly. (DOCX) [file pcbi.1003592.s002.docx]

**Table S1 – Mathews correlation coefficient (MCC) score for the top-performing RF approaches**

Different combinations of three types of nsSNPs are used for each of the three classification problems. RF, RF-SL, RF-SL-2F correspond to the supervised random forest classifier, self-learning random forest classifier, and self-learning random forest classifier using 2 additional features (predictions of effects from the 2-class classifiers), correspondingly.

|  | **Problem 1** | | **Problem 2** | | **Problem 3** | | |
| --- | --- | --- | --- | --- | --- | --- | --- |
| **Method** | **RF** | **RF-SL** | **RF** | **RF-SL** | **RF** | **RF-SL** | **RF-SL-2F** |
| **MCC** | 0.53 | 0.51 | 0.47 | 0.55 | 0.44 | 0.49 | 0.61 |
