# Supplementary material for: Determining Effects of Non-synonymous SNPs on Protein-Protein Interactions using Supervised and Semi-supervised Learning
Source: PLoS Comput Biol. 2014 May 1;10(5):e1003592. doi: 10.1371/journal.pcbi.1003592 (PMC4006705; doi:10.1371/journal.pcbi.1003592)
Supplement: Table S2 — Leave-one-out cross validation results for the top performing supervised and semi-supervised methods trained using more conservative thresholds of ±2.0 kcal/mol. Recall, precision, and f-measure are calculated for each class. Weighted f-measure, fW, average accuracy, Acc, and MCC score are calculated for all classes of a problem. All assessments are based on leave-one-out cross-validation on the labeled dataset. (DOCX) [file pcbi.1003592.s003.docx]

**Table S2 - Leave-one-out cross validation results for the top performing supervised and semi-supervised methods trained using more conservative thresholds of ±2.0 kcal/mol**

Recall, precision, and f-measure are calculated for each class. Weighted f-measure, *f_W_*, and average accuracy, *Acc*, and *MCC* score are calculated for all classes of a problem. All assessments are based on leave-one-out cross-validation on the labeled dataset.

|  |  | **Classifier** | | **Classes** | **Recall** | **Precision** | **f-measure** | ***f_W_*** | ***Acc*** | ***MCC*** |
| --- | --- | --- | --- | --- | --- | --- | --- | --- | --- | --- |
| Problem 1 | Supervised | RF | | Beneficial | 0.35 | 0.89 | 0.51 | 0.93 | 0.94 | 0.41 |
|  |  |  |  | Detrimental | 0.99 | 0.94 | 0.97 |  |  |  |
|  | Semi-supervised | RF-SL | | Beneficial | 0.36 | 1.0 | 0.52 | 0.93 | 0.95 | 0.58 |
|  |  |  |  | Detrimental | 1.0 | 0.94 | 0.97 |  |  |  |
| Problem 2 | Supervised | RF | | Preserving | 0.93 | 0.83 | 0.88 | 0.79 | 0.81 | 0.47 |
|  |  |  |  | Disruptive | 0.48 | 0.71 | 0.57 |  |  |  |
|  | Semi-supervised | | RF-SL | Preserving | 0.92 | 0.85 | 0.88 | 0.81 | 0.82 | 0.51 |
|  |  |  |  | Disruptive | 0.55 | 0.71 | 0.62 |  |  |  |
| Problem 3 | Supervised | RF | | Beneficial | 0.15 | 0.58 | 0.23 | 0.77 | 0.79 | 0.46 |
|  |  |  |  | Detrimental | 0.50 | 0.71 | 0.59 |  |  |  |
|  |  |  |  | Neutral | 0.92 | 0.81 | 0.86 |  |  |  |
|  | Semi-supervised | RF-SL | | Beneficial | 0.15 | 0.70 | 0.24 | 0.78 | 0.79 | 0.47 |
|  |  |  |  | Detrimental | 0.52 | 0.71 | 0.60 |  |  |  |
|  |  |  |  | Neutral | 0.92 | 0.82 | 0.87 |  |  |  |
