# Supplementary material for: Determining Effects of Non-synonymous SNPs on Protein-Protein Interactions using Supervised and Semi-supervised Learning
Source: PLoS Comput Biol. 2014 May 1;10(5):e1003592. doi: 10.1371/journal.pcbi.1003592 (PMC4006705; doi:10.1371/journal.pcbi.1003592)
Supplement: Table S3 — Disease associated mutations studied in the two case studies. Predictions are made using the most accurate classifier for the second 2-class problem, disruptive (D) and preserving (P) PPI mutations. (DOCX) [file pcbi.1003592.s004.docx]

**Table S3 – Disease associated mutations studied in the two case studies.** Predictions are made using the most accurate classifier for the second 2-class problem, disruptive (D) and preserving (P) PPI mutations.

| **Disease** | **Mutation ID** | **Mutation** | **Gene** | **Interaction Partner** | **Binding Site** | **Prediction** |
| --- | --- | --- | --- | --- | --- | --- |
| Breast cancer | rs28903081 | R302H | XRCC3 | RAD51C | No | D |
| Breast cancer | rs28903080 | G271R | XRCC3 | RAD51C | Yes | D |
| Breast cancer | rs77381814 | R243H | XRCC3 | RAD51C | No | P |
| Breast cancer | rs861539 | T241M | XRCC3 | RAD51C | No | P |
| Breast cancer | rs56347206 | R160W | XRCC3 | RAD51C | No | D |
| Breast cancer | rs3212057 | R94H | XRCC3 | RAD51C | No | D |
| Breast cancer | rs28363284 | E233G | RAD51L3 | RAD51C | No | P |
|  |  |  |  | RAD51L1 | No | P |
|  |  |  |  | XRCC2 | No | P |
| Breast cancer | rs28363283 | R232Q | RAD51L3 | RAD51C | Yes | D |
|  |  |  |  | RAD51L1 | Yes | P |
|  |  |  |  | XRCC2 | Yes | P |
| Breast cancer | rs28363282 | A225T | RAD51L3 | RAD51C | Yes | P |
|  |  |  |  | RAD51L1 | Yes | P |
|  |  |  |  | XRCC2 | Yes | P |
| Breast cancer | rs80116829 | A190T | RAD51L3 | RAD51C | No | P |
|  |  |  |  | RAD51L1 | No | P |
|  |  |  |  | XRCC2 | No | P |
| Breast cancer | rs4796033 | R165Q | RAD51L3 | RAD51C | No | P |
|  |  |  |  | RAD51L1 | No | P |
|  |  |  |  | XRCC2 | No | P |
| Breast cancer | rs55942401 | E157D | RAD51L3 | RAD51C | No | P |
|  |  |  |  | RAD51L1 | No | P |
|  |  |  |  | XRCC2 | No | P |
| Breast cancer | rs56026142 | V66M | RAD51L3 | RAD51C | Yes | P |
|  |  |  |  | RAD51L1 | Yes | P |
|  |  |  |  | XRCC2 | Yes | P |
| Diabetes | rs1169288 | I27L | HNF1A | PCBD1 | No | P |
| Diabetes | rs115117837 | R88Q | PCBD1 | HNF1A | No | P |
|  |  |  |  | PCBD1 | No | P |
| Diabetes | rs104894177 | C82R | PCBD1 | HNF1A | No | P |
|  |  |  |  | PCBD1 | No | P |
| Diabetes | rs11554325 | R31C | PCBD1 | HNF1A | No | P |
|  |  |  |  | PCBD1 | No | P |
